# Professor Eve’s Card

**Published:** 1851-01

**Authors:** 


*Professor Eve's Card.*—We published in the December number of this *Journal*, Professor Eve's Card, on the subject of the Report on Surgery, to be made by him, to the American Medical Association, next May. By a typographical blunder, he was made to call himself Chairman of the American Medical Association, instead of Chairman of the Committee of Surgery of that body. It is such an obvious error, that our confrêres of the press, will find no difficulty, we hope, in correcting it. B.

